# Supplementary material for: Proteomic analysis of young and old mouse hematopoietic stem cells and their progenitors reveals post-transcriptional regulation in stem cells
Source: eLife. 2020 Nov 25;9:e62210. doi: 10.7554/eLife.62210 (PMC7688314; doi:10.7554/eLife.62210)
Supplement: Transparent reporting form [file elife-62210-transrepform.pdf]

Westbrook Centre, Milton Road
Cambridge CB4 1YG
UK

P 01223 855340
W elifesciences.org
T @elife

eLife's transparent reporting form

We encourage authors to provide detailed information *within their submission* to facilitate the interpretation and replication of experiments. Authors can upload supporting documentation to indicate the use of appropriate reporting guidelines for health-related research (see EQUATOR Network), life science research (see the BioSharing Information Resource), or the ARRIVE guidelines for reporting work involving animal research. Where applicable, authors should refer to any relevant reporting standards documents in this form.

If you have any questions, please consult our Journal Policies and/or contact us: editorial@elifesciences.org.

Sample-size estimation

- You should state whether an appropriate sample size was computed when the study was being designed
- You should state the statistical method of sample size computation and any required assumptions
- If no explicit power analysis was used, you should describe how you decided what sample (replicate) size (number) to use

Please outline where this information can be found within the submission (e.g., sections or figure legends), or explain why this information doesn't apply to your submission:

For the adult samples, our 6 replicates are a combination of 3 biological pooled cohorts of mice as well as technical duplicate of the sample being run on the mass spectrometer. This is above and beyond what is typically required (2 biological replicates), but we wanted to try to capture as many proteins as possible. The diminishing return of proteomic yield beyond 6 replicates is shown in figure 1 and described in "Optimization of sample preparation for low numbers of rare cells" within results. For our aged data we had 4 replicates, 2 biological pooled cohorts of mice in technical duplicate. We used equal numbers of male and female mice and detail accordingly throughout the manuscript. For non mass-spectrometry experiments we indicate the number of mice in both the methods as well as figure legends.

Replicates

- You should report how often each experiment was performed
- You should include a definition of biological versus technical replication
- The data obtained should be provided and sufficient information should be provided to indicate the number of independent biological and/or technical replicates
- If you encountered any outliers, you should describe how these were handled
- Criteria for exclusion/inclusion of data should be clearly stated
- High-throughput sequence data should be uploaded before submission, with a private link for reviewers provided (these are available from both GEO and ArrayExpress)

Please outline where this information can be found within the submission (e.g., sections or figure legends), or explain why this information doesn't apply to your submission:

eLife Sciences Publications, Ltd is a limited liability non-profit non-stock corporation incorporated in the State of Delaware, USA, with company number 5030732, and is registered in the UK with company number FC030576 and branch number BR015634 at the address Westbrook Centre, Milton Road Cambridge CB4 1YG, UK | March 2019

We detail explicitly that we use a combination of biological and technical replicates for our mass spectrometry experiments in both the main text as well as methods section. For non-mass spectrometry experiments we highlight the number of mice used for each analysis. All data is available through the PRIDE repository as indicated in the methods section of the manuscript. All processed data is also included as a supplemental data excel spreadsheet for ease of access. Representative FACS plots for all FACS experiments are included in Figure Supplements. We chose to exclude zero values from our mass spectrometry data, unless it was never detected in any replicate, in which case we denote as N.D. (not detected). We discuss this is in our manuscript/methods, and it is standard in mass spectrometry data presentation. We choose to use the words 'detectable', 'detected'…etc in order to highlight that these are proteins that are unable to be seen by the instrumentation – it is not necessarily an absolute statement that the protein itself is absent.

Statistical reporting

- Statistical analysis methods should be described and justified
- Raw data should be presented in figures whenever informative to do so (typically when N per group is less than 10)
- For each experiment, you should identify the statistical tests used, exact values of N, definitions of center, methods of multiple test correction, and dispersion and precision measures (e.g., mean, median, SD, SEM, confidence intervals; and, for the major substantive results, a measure of effect size (e.g., Pearson's r, Cohen's d)
- Report exact p-values wherever possible alongside the summary statistics and 95% confidence intervals. These should be reported for all key questions and not only when the p-value is less than 0.05.

Please outline where this information can be found within the submission (e.g., sections or figure legends), or explain why this information doesn't apply to your submission:

When representing the proteomic data all individual non-zero values are shown as data points with appropriate N indicated. For non-mass spectrometry data, all individual raw data is presented in graphs as data points with N indicated.

Which statistical tests are used is described in detail in the methods, including information on definitions of center, multiple test correction, dispersion, and correlation tests. All p-values are reported in the figure legends. Error bars are calculated as standard error to the mean as indicated in each figure legend.

(For large datasets, or papers with a very large number of statistical tests, you may upload a single table file with tests, Ns, etc., with reference to sections in the manuscript.)

Group allocation

- Indicate how samples were allocated into experimental groups (in the case of clinical studies, please specify allocation to treatment method); if randomization was used, please also state if restricted randomization was applied
- Indicate if masking was used during group allocation, data collection and/or data analysis

Please outline where this information can be found within the submission (e.g., sections or figure legends), or explain why this information doesn't apply to your submission:

Within our methods and manuscript text we highlight the rationale behind the number of experimental replicates, the number of cells/mice used, their gender and, in the case of mass spectrometry data, efforts to normalize the contribution of cells from each mouse to a biological cohort. Equal numbers of male and female mice were used throughout as indicated. No masking was used during these studies or analysis.

Additional data files ("source data")

- We encourage you to upload relevant additional data files, such as numerical data that are represented as a graph in a figure, or as a summary table
- Where provided, these should be in the most useful format, and they can be uploaded as "Source data" files linked to a main figure or table
- Include model definition files including the full list of parameters used
- Include code used for data analysis (e.g., R, MatLab)
- Avoid stating that data files are "available upon request"

Please indicate the figures or tables for which source data files have been provided:

The two primary data files are proteomic and transcriptomic data used throughout the entire manuscript, both of which are part of our submission in a table format. Raw mass spectrometry data has been uploaded to the PRIDE repository as is indicated in the manuscript.

The link for code is indicated in the "Data Code and Availability" section of the methods
